# Knockdown of TFIIS by RNA silencing inhibits cancer cell proliferation and induces apoptosis

**DOI:** 10.1186/1471-2407-8-133

**Published:** 2008-05-12

**Authors:** Kyle Hubbard, Jennifer Catalano, Raj K Puri, Averell Gnatt

**Affiliations:** 1 Pharmacology and Experimental Therapeutics, University of Maryland School of Medicine, Baltimore, MD, 21201, USA; 2 Center for Biologics and Evaluation Research, United States Food and Drug Administration, Bethesda, MD, 20892, USA; 3 Marlene and Stewart Greenebaum Cancer Center, University of Maryland Baltimore, Baltimore, MD, 21201, USA

## Abstract

**Background:**

A common element among cancer cells is the presence of improperly controlled transcription. In these cells, the degree of specific activation of some genes is abnormal, and altering the aberrant transcription may therefore directly target cancer. TFIIS is a transcription elongation factor, which directly binds the transcription motor, RNA Polymerase II and allows it to read through various transcription arrest sites. We report on RNA interference of TFIIS, a transcription elongation factor, and its affect on proliferation of cancer cells in culture.

**Methods:**

RNA interference was performed by transfecting siRNA to specifically knock down TFIIS expression in MCF7, MCF10A, PL45 and A549 cells. Levels of TFIIS expression were determined by the Quantigene method, and relative protein levels of TFIIS, c-myc and p53 were determined by C-ELISA. Induction of apoptosis was determined by an enzymatic Caspase 3/7 assay, as well as a non-enzymatic assay detecting cytoplasmic mono- and oligonucleosomes. A gene array analysis was conducted for effects of TFIIS siRNA on MCF7 and MCF10A cell lines.

**Results:**

Knockdown of TFIIS reduced cancer cell proliferation in breast, lung and pancreatic cancer cell lines. More specifically, TFIIS knockdown in the MCF7 breast cancer cell line induced cancer cell death and increased c-myc and p53 expression whereas TFIIS knockdown in the non-cancerous breast cell line MCF10A was less affected. Differential effects of TFIIS knockdown in MCF7 and MCF10A cells included the estrogenic, c-myc and p53 pathways, as observed by C-ELISA and gene array, and were likely involved in MCF7 cell-death.

**Conclusion:**

Although transcription is a fundamental process, targeting select core transcription factors may provide for a new and potent avenue for cancer therapeutics. In the present study, knockdown of TFIIS inhibited cancer cell proliferation, suggesting that TFIIS could be studied as a potential cancer target within the transcription machinery.

## Background

An underlying mechanism of breast and other cancers involves aberrant transcription with numerous genes up or down-regulated [1-6]. It is reasonable to assume that further perturbing the improper transcription occurring in cancer cells could result in cancer cell death. Transcription, however, is a fundamental cellular process, and its targeting may affect non-cancerous cells. Nonetheless, it has been proposed that targeting transcription is possible and challenges in attaining cancer specificity can be overcome [7].

RNA Polymerase II (RNAP) is the multisubunit enzyme responsible for generating all mRNA in eukaryotic cells [8,9]. All stages of regulation of RNAP could be potential targets for cancer therapy including initiation and/or termination of the transcription process as well as elongation of the mRNA and termination. Another target could include components of the machinery involved in chromatin remodeling and the positioning of nucleosomes, structures composed of DNA wrapped around a histone protein core [10,11]. Chromatin remodeling is important in allowing RNAP access to DNA such that histone deacetylase (HDAC) inhibitors, which modulate nucleosome structure, are effective as anticancer agents [12,13].

We tested knockdown of several components of the transcription machinery for effects on cancer cells and found TFIIS knockdown of interest for further analysis. During transcript elongation, RNAP can arrest on specific DNA sequences including Poly T stretches, unable to complete the synthesis of mRNA [14,15]. When RNAP arrests, the active site disengages from the 3' end of the transcript and repositions itself over an internal phosphodiester bond and is therefore incapable of adding ribonucleotide substrates [16]. TFIIS reactivates arrested transcription by stimulating RNAP endonucleolytic cleavage of the transcript [17,18]. Once cleavage of the RNA is completed, the active site is correctly positioned at the new 3'-end of the RNA chain allowing for chain extension. As a result, TFIIS induced readthrough of arrest sites produces both a 7–9 base RNA cleavage product and a full-length readthrough product.

However, alternate mechanisms exist to deal with arrested transcription. Transcription elongation factors such as TFIIF, ELL and Elongin are able to suppress arresting so that there is no need for reactivation [19]. Alternatively, RNAP in an arrested complex can be subject to degradation by the ubiquitin/proteosome pathway [20].

Initially we tested effects of siRNA knockdown of several transcription factors. TFIIS presented the best case for further analysis and the TFIIS data is presented herein. Our evidence indicates that TFIIS knockdown inhibits cell proliferation and induces apoptosis in cancer cells.

## Methods

### Cell Culture

MCF7 and PL45 cells were grown in DMEM + 10% Fetal Bovine Serum (FBS) + 1% Penicillin/Streptomycin (Pen/Strep, Invitrogen). A549 and PC-3 were grown in F-12 (Ham's) Media + 10% Fetal Bovine Serum (FBS) + 1% Penicillin/Streptomycin (Pen/Strep). MCF10A cells were grown and plated in MEGM (Clonetics, CC-3150) supplemented with Bovine Pituitary Extract (BPE), human Endothelial Growth Factor (hEGF), hydrocortisone, GA-1000 and insulin. For assays Pen/Strep was omitted. All cell lines were obtained from the ATCC.

### siRNA Proliferation Assay

siRNAs targeting different regions of human TFIIS are listed in Table 1. siRNA was generated using the *Silencer *siRNA Construction Kit (Ambion #1620) and transfected into cells using Lipofectamine 2000 (Invitrogen #11668-019). Cell proliferation assays employ the MTS assay (Promega, WI). Before transfection, cell lines were seeded in 96-well plates at a concentration of 2000 cells/well. Plates were incubated for 24 hrs at 37°C. Lipofectamine 2000 Transfection Reagent (Invitrogen) was mixed with 0.1, 0.5, 1.0, and 5.0 nM Eg5 siRNA (positive control), S-siRNA negative control (sGAPDH, Ambion), or each of the TFIIS siRNAs in Opti-MEM I Reduced Serum Medium (Invitrogen #31985-070). Cells were transfected using the Opti-MEM containing the transfection reagent and the siRNA plus their respective growth medium without antibiotic. Cells were incubated for 72 hrs at 37°C. Vehicle containing Lipofectamine 2000 alone was applied to cells and viable cells considered 100% survival. Assays were performed in quadruplicate with a minimum of two different experiments. For cell viability, media was replaced with MEM without Phenol Red, and MTS reagent was added and allowed to incubate for 2 hrs, at which time the colorimetric signal was determined, and data analyzed to obtain the mean and standard error. Graphs were plotted using Prism (Graphpad Software, Inc., CA). For the proliferation and caspase assays, a one-way ANOVA was performed for all data generated indicating the overall significance of the data. However, data which was not significant (p > 0.05), was noted in the text of the result section.

**Table 1:** siRNA's Employed in Knockdown Experiments.

**Gene**	**Sense Strand**	**Antisense Strand**	**mRNA Target**
TFIIS #1	aatgctattcgcaagcagagt	aaactctgcttgcgaatagca	aaugcuauucgcaagcagagu
TFIIS #2	aacaggggatgactacattgc	aagcaatgtagtcatcccctg	aacaggggaugacuacauugc
TFIIS #3	aagagatgcggaaaaacttga	aatcaagtttttccgcatctc	aagagaugcggaaaaacuuga

Sense and antisense template strands shown also contained a cctgtctc T7 promoter primer sequence on the 3' terminal. Once the individual template strands had been transcribed, they were hybridized to form the 21-nt double stranded short interfering RNA (siRNA).

### Knockdown of mRNA by siRNA

Knockdown of mRNA levels was assessed by the Quantigene method as suggested by company protocols (Panomics, Fremont, CA). Controls are listed in materials and methods above. mRNA levels were directly quantified from cells grown in 96-well plates. The assay employed 5 nM TFIIS siRNA#2 and expression was normalized to cell number as determined by the MTS assay. Cells treated with 5 nM S-siRNA were set at 100% cell proliferation.

### C-ELISA

Cells were plated in 96-well plates in quadruplicate. After a 72-hour exposure to siRNA, media was removed and 3.7% formaldehyde was added as a fixative and allowed to incubate for 10 minutes. After fixation, cells were washed with PBS-0.5% Tween (PBS-T) three times for five minutes each. Cells were then blocked in 5% non-fat milk + 1% Bovine Serum Albumin (BSA) in PBS-0.5% Tween (PBS-T) for one hour at room temperature. Blocking reagent was removed and primary antibody diluted 1:500 in block buffer was added and left to incubate for one hour at room temperature. Primary antibody was removed, followed by three washes in PBS-T for 1 minute each. Secondary antibody conjugated to HRP (KPL cat#074-1806) was added at a dilution of 1:1000 in block buffer and left to incubate for 1 hour at room temperature. Three PBS-T washes followed, each for 1 minute. During this time, a chemiluminescent substrate (Pierce, cat #34095) was prepared and added to the cells and chemiluminescence read in a Wallac Victor 2 plate reader (Perkin Elmer; Waltham, MA). Antibodies employed in the assay included c-myc (Sigma cat#M4439), TFIIS (BD Biosciences cat #611205), GAPDH (Calbiochem cat#CB1001), and p53 (Sigma cat#P6874).

### Western Analysis

Standard western analysis was performed employing antibodies for TFIIS (BD Biosciences cat #611205) and for α-tubulin (Santa Cruz Biotechnology cat# sc-58667). 10 nM siRNA was employed and cells collected at 48 hours to allow for enough starting material. 2.5 × 10^6 ^cells/plate were plated in 10 cm plates and transfected with siRNA. After 48 hours, cells were washed in PBS and adherent cells were collected. RIPA buffer supplemented with 1:100 protease inhibitors was added for 30 minutes and after centrifugation, protein in the supernatant was collected and stored at -80°C. Protein was loaded onto a 10% SDS-PAGE gel and transferred overnight to nitrocellulose at 4°C.

Blocking of the nitrocellulose was performed with 5% non-fat milk in PBS-0.5% Tween (PBS-T) for one hour at room temperature. Primary antibody was diluted 1:1000 in 5% non-fat milk PBS-T incubated for one hour at room temperature. The blot was washed three times in PBS-T for 10 minutes each. Secondary antibody conjugated to HRP (KPL cat#074-1806) was added at a dilution of 1:5000 in 5% non-fat milk PBS-T and incubated for 45 minutes at room temperature. The filter was washed three times with PBS-T washes for 10 minutes each wash and detection was performed employing an ECL Detection Reagent (Amersham). The filter was exposed to film and images were scanned and quantified by Imagequant (BioRad).

### Apoptosis

Apoptosis was tested by an enzymatic and nonenzymatic assay. Each assay was performed in quadruplicate with a minimum of two independent experiments. For the enzymatic assay, cells were treated as indicated for the MTS proliferation assay with MTS being substituted by the Caspase Glo-3/7 Assay reagent (Promega cat #G8091) and plates read as instructed by the manufacturer. Values for caspase activity were divided by the amount of viable cells as represented by MTS values and normalized to similar data from vehicle treated cells. The data was analyzed to obtain the mean and standard error, and graphs were plotted using Prism (Graphpad Software, Inc., CA).

For the nonenzymatic assay we employed the Cell Death Detection ELISA^PLUS ^Assay (Roche Cat. No. 11774425001), which determines the level of cytoplasmic mono- and oligonucleosomes induced by apoptosis. Cells were treated with 5 nM TFIIS siRNA #2, or 5 nM S-siRNA in 6-well plates, incubated at 72°C for 72 hours, and the assay completed as recommended by the manufacturer. Enrichment of mono- and oligonucleosomes was determined by dividing TFIIS siRNA values by those of cells treated with 5 nM S-siRNA. The data was analyzed to obtain the mean and standard error, and graphs were plotted using Prism (Graphpad Software, Inc., CA)

### Gene Arrays

#### I) Experimental Design

A total of two experiments were completed with 4 chips each, so that the microarray replicates were performed in duplicate for a total of 8 chips. Details of the experimental design can be found in Table 2.

**Table 2:** Gene Array: Experimental Design

Chip number	Cy 3 label	Cy 5 label
1	Reference: MCF7 RNA with scrambled control sequence	MCF10A w/scrambled control sequence
2	Reference: MCF7 RNA with scrambled control sequence	MCF10A w/TFIIS siRNA sequence
3	Reference: MCF7 RNA with scrambled control sequence	MCF7 w/scrambled control sequence
4	Reference: MCF7 RNA with scrambled control sequence	MCF7 w/TFIIS siRNA sequence
5	Reference: MCF7 RNA with scrambled control sequence	MCF10A w/scrambled control sequence
6	Reference: MCF7 RNA with scrambled control sequence	MCF10A w/TFIIS siRNA sequence
7	Reference: MCF7 RNA with scrambled control sequence	MCF7 w/scrambled control sequence
8	Reference: MCF7 RNA with scrambled control sequence	MCF7 w/TFIIS siRNA sequence

#### II) Generation of Probes

Arrays were preformed in duplicate as previously described [21]. Oligonucleotide glass array containing a total of 16,659 seventy-mer oligonucleotides chosen from 750 bases of the 3' end of each ORF (Operon Inc. Valencia, CA) spanning approximately 50% of the human genome and produced spotting oligonucleotides on poly-L-lysine coated glass slides by Gene Machines robotics (Omnigrid, San Carlos, CA). Total RNA was isolated from TFIIS#2 and S-siRNA treated MCF7 and MCF10A cell lines using the Trizol reagent (Invitrogen). The precise probe and control employed for each chip is provided in additional file 1.

Labeled cDNA was produced as previously described [22]. Briefly, 5 μg of total RNA was dissolved in 12 μl of DEPC water and incubated at 70°C for 5 minutes along with 1 μl of aminoallyl-oligo dT (5' amino-modified) primer and chilled for 3 minutes. Then, 1.5 μl AffinityScript reverse transcriptase (Stratagene, La Jolla, CA), 2 μl of 10× first strand buffer supplied with the reverse transcriptase, 1.5 μl dNTP mix (10 mM dATP, dGTP, and dCTP, 6 mM dTTP, 4 mM amino-allyl dUTP), and 2 μl of 0.1 M DTT were added and incubated for 90 minutes at 44°C. After incubation, the volume of the reaction mixture was brought to 60 μl with 40 μl DEPC water. 300 μl of Binding buffer PB was added to the coupled cDNA, and the mixture applied to a MinElute column (Qiagen, Valencia, CA), centrifuged for 1 minute at maximum speed and washed twice with 400 μl of buffer PE. The center of the membrane was incubated at room temperature for 1 min with 10 μl elution buffer for 1 min and centrifuged for 1 min at max speed to elute the cDNA probe. Finally, 5 μl of 0.4 M NaHCO_3 _coupling buffer and 10 pmol of either Cy3 or Cy5 dye dissolved in 5 μl DMSO was added to the control (MCF7 or MCF10A cells treated with S-siRNA) or experimental cDNAs (MCF7 and MCF10A treated with TFIIS siRNA). The reaction was incubated at room temperature in the dark for 90 minutes. The volume was then brought to 60 μl by the addition of 50 μl DEPC water and cDNA was purified by MinElute column as described above. Finally, the Cy3 and Cy5 labeled cDNAs were mixed.

#### III) Hybridization

Samples were heated at 100°C for 2 minutes and 25 μl hybridization buffer (Slide Hyb#1 Catalog #8861, Applied Biosystems, Foster City, CA) was added to the labeled probes. Then, 42 μl of probe was added to the arrays covered with a MAUI^® ^Mixer FL hybridization chamber (Biomicrosystems, Salt Lake City, UT). Slides were placed into a 4-bay MAUI^® ^Hybridization System Instrument overnight (14–16 hours) at 42°C, then washed for 5 minutes each in 1× SSC and 0.1× SSC and spin-dried at 500 ×*g*.

#### IV) Data filtration, Normalization, and Analysis

Microarray slides were scanned in Cy3 (532 nm) and Cy5 (635 nm) channels using Axon GenePix 4000B scanner (Axon Instruments, Inc., Foster City, CA) with a 10-micron resolution and exported as TIFF files to GenePix Pro 3.0 software for image analysis. The raw images were collected at 16-bit/pixel resolutions with 0 to 65,535 count dynamic range. Background values were not subtracted. The resulting GenePix data was imported into ArrayTrack Software (Food and Drug Administration, National Center for Toxicological Research) [23]. The data was normalized using the software's LOWESS and linear normalization methods. A t-test was performed on the scrambled control vs. the TFIIS siRNA treated groups for each cell line to get the list of differentially expressed genes (Test 1 = MCF7 scrambled control vs. MCF7 treated; Test 2: MCF10A scrambled control vs. MCF10A siRNA treated). A Bonferroni adjusted *p*-value was too stringent and yielded no significant genes. A *p*-value of 0.05 was used while adding a more stringent fold-change criteria (1.3). Using both a *p*-value and fold change cut-offs are in accordance with the results of reproducibility found by the Microarray Quality Control Consortium (Nature Biotechnology, September 2006 Vol. 24 No. 9 issue). The lists were then imported into the Ingenuity database for pathway analysis (Ingenuity, Redwood City, CA).

## Results

### Differential effects of TFIIS knockdown on MCF7 and MCF10A cell lines

**Figure 1:**
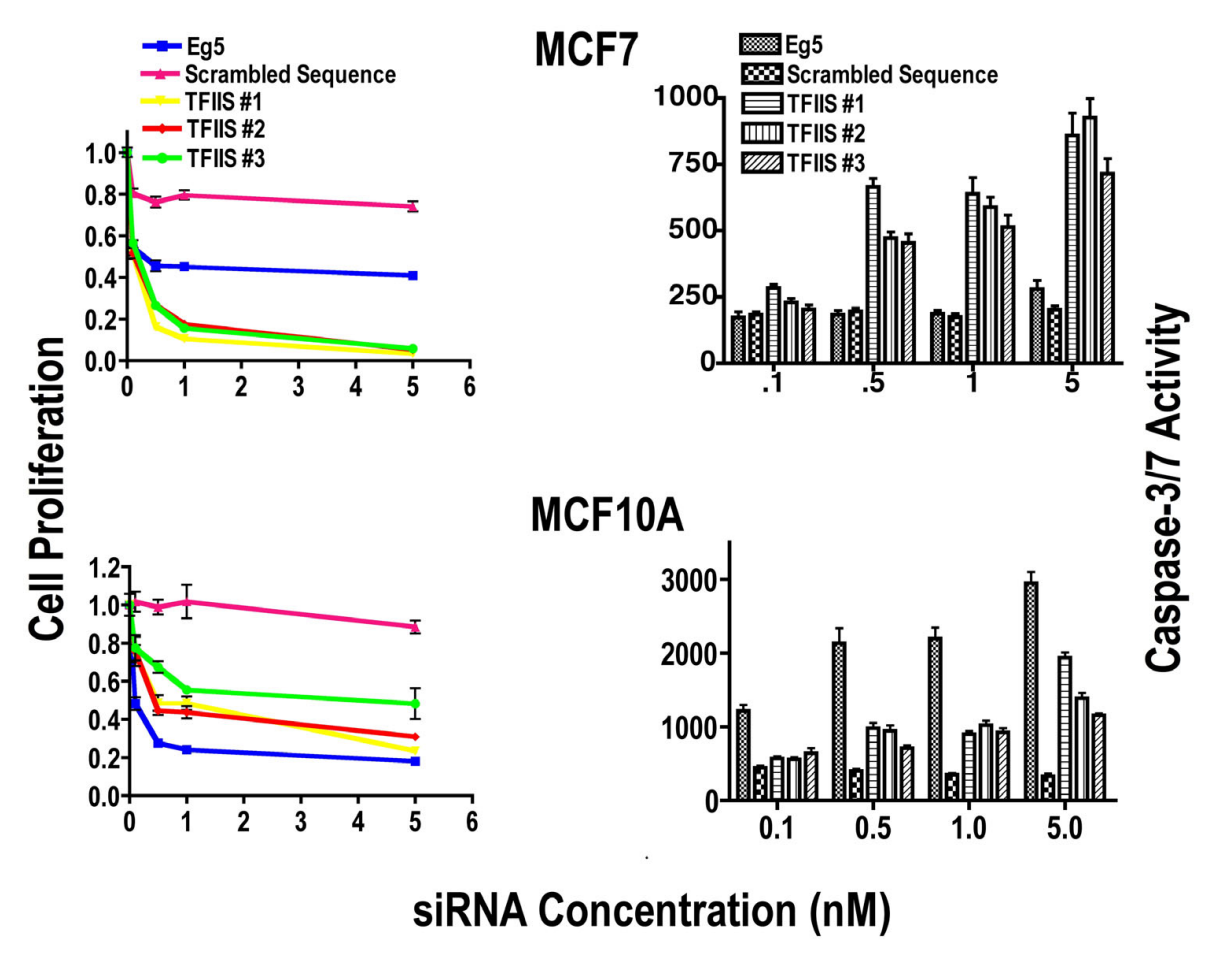
**Effects of TFIIS siRNA on Cell Proliferation**. MCF7 and MCF10A cell lines were subjected to siRNA directed at TFIIS as listed in Table 1, scrambled siRNA (S-siRNA) or Eg5 and MTS values determined 72 hours after transfection. Cell proliferation represents the fraction of viable cells compared to cells transfected with vehicle alone (1 = 100% viable cells when treated with vehicle alone). Standard error of the mean is indicated as bars in the figure. Caspase-3/7 activity is also depicted and normalized to cell number represented by MTS values. Total activity was then normalized to activity in cells treated with vehicle alone.

We determined effects of TFIIS siRNA on the proliferation of both cancerous MCF7 and non-cancerous MCF10A breast cell lines (Figure 1). For validation purposes three different siRNAs targeting different regions of human TFIIS lacking homology to other human genomic sequences were employed (Table 1). A scrambled GAPDH siRNA (S-siRNA, Ambion, cat #4605) was employed as a negative control. Eg5 siRNA was employed as positive control. Eg5 is required for the formation of the bipolar spindle and chromosome separation [24,25] and has been determined to be an effective control for siRNA experiments [25]. Eg5 is also a cancer target [26] so that it provides for an effective comparison with transcription factor siRNAs. Effects of Eg5 knockdown are known to vary depending on cell line, and can either inhibit cell division preventing proliferation, or induce apoptosis [27].

We also determined if reduced proliferation by TFIIS siRNA occurs concomitantly with activation of caspases 3 and 7 by employing an assay that detects the sum of caspases 3 and 7 activities. MCF7 cells lack functional caspase 3 so that the caspase-3/7 activity assay in those cells represents caspase 7 activity alone [28]. Detection of caspase-3/7 activity was employed since caspase 7 is a critical mediator of mitochondrial events of apoptosis [29].

In MCF7 cells, all three TFIIS siRNAs induced a dramatic concentration-dependent reduction in cell proliferation and an increase in caspase-3/7 activity (Figure 1). A one-way ANOVA confirms that all differences in cell proliferation and caspase-3/7 activity between 5 nM S-siRNA and 5 nM TFIIS siRNAs were significant (p < 0.001). However, there was no significant difference in caspase-3/7 activity between S-siRNA and Eg5 treated cells. Compared to MCF7, TFIIS siRNA treated MCF10A cells displayed significantly less growth inhibition. The overall higher values of caspases 3/7 activity in the MCF10A cells are to be expected because of the absence of caspase 3 activity in MCF7 cells [28]. In both MCF7 and MCF10A, the negative control S-siRNA had relatively limited off-target effects on cell proliferation and the positive Eg5 siRNA control was effective in reducing proliferation as expected. However, Eg5 siRNA inhibition of proliferation was greater in MCF10A than in MCF7 even at relatively lower concentrations of siRNA. This is consistent with variable effects of Eg5 knockdown on different cell lines [27].

**Figure 2:**
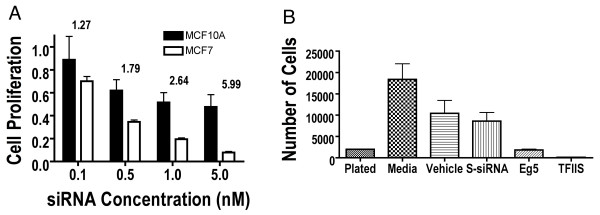
**TFIIS siRNA is specific for cancer cells and causes cell death**. (A) MCF10A (Black) and MCF7 (White) cell lines were subjected to siRNA directed at TFIIS employing siRNA#3 and proliferation determined 72 hours after treatment. siRNA concentrations and the fraction of cell proliferation normalized to S-siRNA treated cells are indicated (1 = 100% viable cells when treated with S-siRNA). The ratio of cell proliferation between MCF10A and MCF7 cells is shown above the bars. (B) The bar graph represents the number of trypan blue excluded cells. Plated, represents the number of cells initially seeded. Media represents the number of cells when employing unmodified media. Also listed is the number of viable cells after treatment of MCF7 with lipid transfer vehicle identified as "Vehicle" TFIIS siRNA, S-siRNA or Eg5 siRNA. Data points represent mean and standard error. Proliferation was determined 72 hours after siRNA treatment.

The ratio of proliferation of MCF10A to MCF7 when treated with TFIIS siRNA normalized to S-siRNA control proved to be significant and positive (Figure 2A). At the highest concentration of TFIIS siRNA#3 (5 nM), a 6-fold anti-proliferative ratio existed between non-cancer and cancer cells (4.9-fold for TFIIS siRNA#2). The anti-proliferative effects of TFIIS siRNA, on MCF7 cells was also associated with cell death as determined by a trypan blue exclusion cell viability assay (Figure 2B). Initially, 2,000 cells were plated per well. Seventy-two hours after treatment with 5 nM TFIIS siRNA#2, only 140 (± 26) MCF7 cells survived whereas treatment with vehicle or S-siRNA resulted in 10,425 (± 3,012) or 8,600 (± 1,998) viable cells respectively. Essentially, MCF7 cannot survive treatment with TFIIS siRNA.

### TFIIS siRNA reduces proliferation of cancer cells of different origin

**Figure 3:**
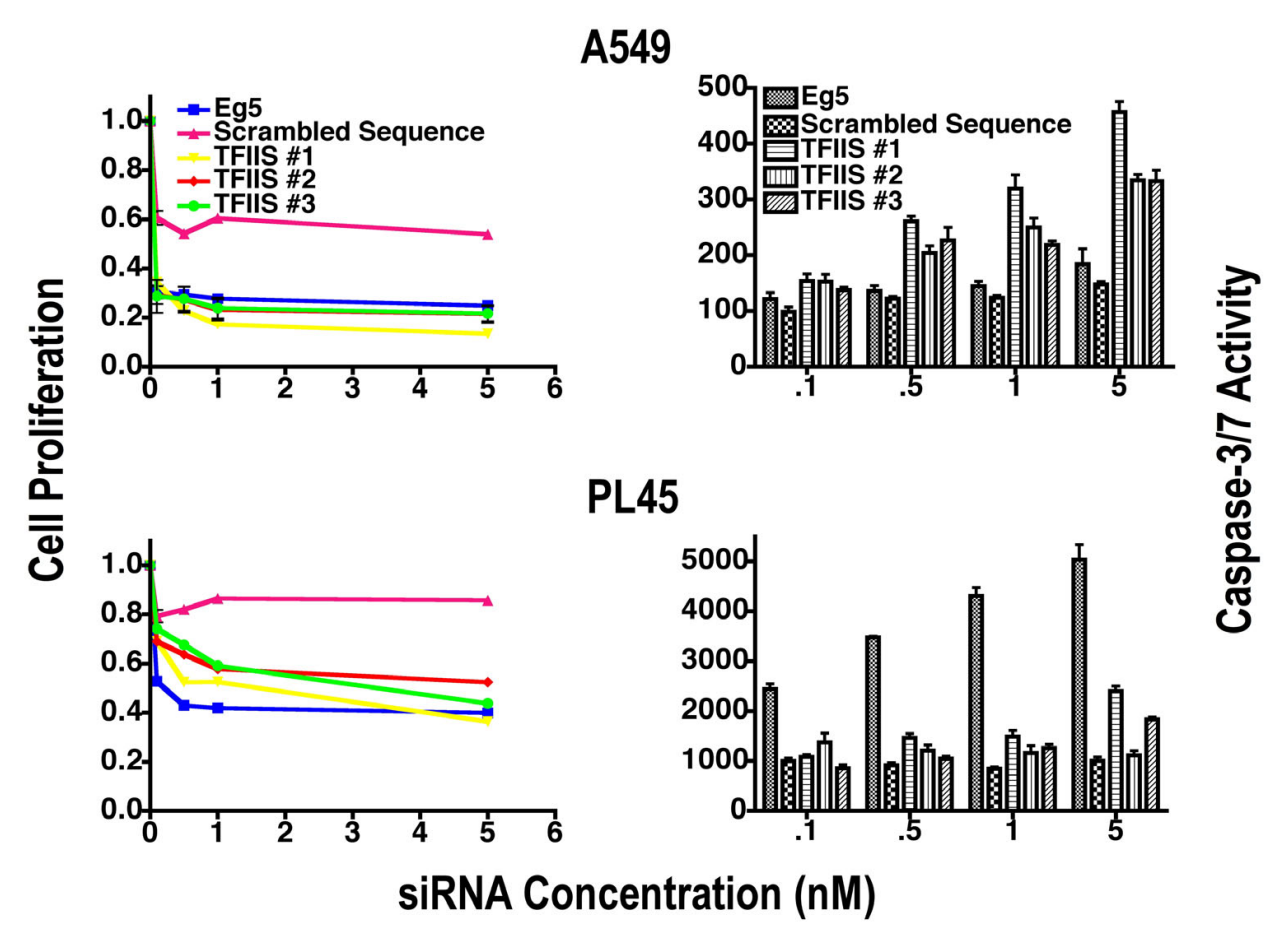
**Effects of TFIIS siRNA on proliferation of lung A549 and pancreatic PL45 cells**. A549 and PL45 cell lines were subjected to siRNA directed at TFIIS #1–3, Scrambled siRNA (S-siRNA), or Eg5. Cell proliferation represents the fraction of viable cells compared to cells transfected with vehicle alone (1 = 100% viable cells). Caspase-3/7 activity is also depicted and normalized to cell number represented by MTS values. Total activity was then normalized to activity in cells treated with vehicle alone. TFIIS siRNA #2 nearly perfectly overlays TFIIS siRNA #1 in MCF7 cells and is therefore mostly not visible. Data points represent mean and standard error. The assay was performed 72 hours after initial siRNA transfection.

It was not initially clear if TFIIS siRNA would have a cancer type specific effect, so that we also tested lung, A549 and pancreas PL45 cell lines in our proliferation and caspase 3/7 assays (Figure 3). Both A549 and PL45 cell lines proved susceptible to TFIIS siRNA in a concentration-dependent manner with A549 displaying a greater susceptibility than PL45 (Figure 3). Inhibition of proliferation as a result of the positive control Eg5 siRNA indicated effective transformation and all three TFIIS siRNAs provided similar inhibition profiles in accord with specificity of targeting. However, S-siRNA itself inhibited proliferation in A549 cells to a greater extent than for MCF7, MCF10A or PL45, indicating that some cell lines may be more susceptible to non-specific effects of siRNA than others.

When comparing TFIIS siRNA proliferation profiles of MCF7, A549, and PL45 to the noncancerous cell line MCF10A, MCF7 displayed the greatest differential. A549 cells presented a significant increased level of inhibition of proliferation of A549 compared to MCF10A only at low siRNA concentration, and there was no significant difference between PL45 and MCF10A cells.

It is also important to note that 5 nM TFIIS siRNA induced caspase-3/7 activation for all cell lines tested. The only exception was for PL45 cells, in that a one-way ANOVA indicates no significant caspase 3/7 activity at lower TFIIS siRNA concentrations and a minimal signal relative to S-siRNA at 5 nM TFIIS siRNAs #1 and #3 (p < 0.001 and p < 0.05 respectively). Eg5 siRNA induced dramatic caspase 3/7 activity in MCF10A and PL45 and minimal increases in MCF7 and A549 cell lines, in accord with differential effects of Eg5 inhibition on diverse cell lines [27].

**Figure 4:**
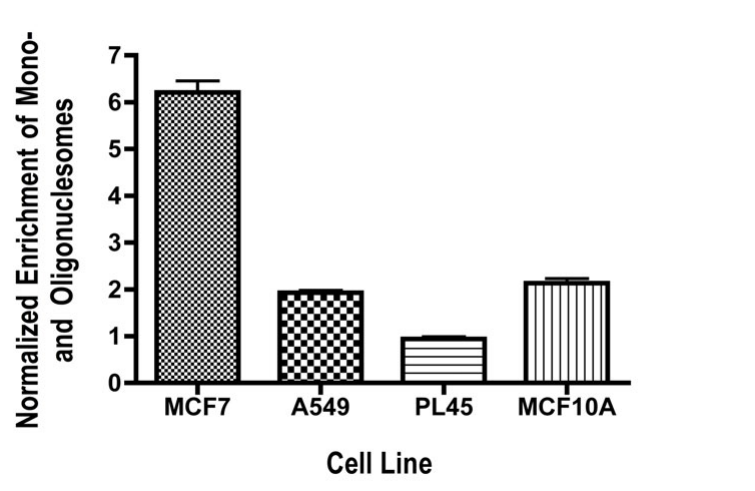
**Enrichment of Mono- and Oligonucleosomes after introduction of TFIIS siRNA**. MCF7, PL45, A549 and MCF10A cells were treated with 5 nM TFIIS #2 siRNA and S-siRNA. Mono- and oligonucleosome values were normalized to cells treated with S-siRNA, so that a value of one represents a lack of apoptosis. Assays were performed for 72 hours.

To further validate the enzymatic caspase 3/7 apoptosis data, we employed a non-enzymatic assay to detect cytoplasmic mono- and oligonucleosomes, which are consequences of apoptosis (Figure 4). In the assay MCF7 cells treated with 5 nM TFIIS siRNA #2 displayed a robust increase in apoptotic activity while A549 and MCF10A cells showed a modest two-fold increase compared to S-siRNA. Apoptosis was not statistically significant in PL45 cells. This is in good accord with the caspase data, where TFIIS siRNA induced pronounced dose-dependent caspase activity in MCF7 cells, yet barely any activity in PL45 cells. It also correlates well with the high sensitivity and effective elimination of MCF7 cells as a result of TFIIS siRNA.

### TFIIS siRNA effects are due to a reduction in TFIIS mRNA and protein levels

We determined TFIIS mRNA levels employing TFIIS siRNA#2 and the Quantigene method as suggested by the manufacturer (Panomics [30]). TFIIS mRNA levels were 31.0 ± 1.6% for MCF7 and 45.4 ± 5.0% for MCF10A when compared to the 100% levels set for S-siRNA treated cells.

To determine TFIIS protein levels in 5 nM TFIIS siRNA treated MCF7 and MCF10A cells, we employed a C-ELISA assay and GAPDH antibodies as control. GAPDH proved to be a valid control as GAPDH and cell proliferation were linearly correlated (supplemental data). TFIIS protein levels in MCF7 cells were 0.43 ± 0.1 and 0.28 ± 0.07 for MCF10A when compared to TFIIS levels set at 1 for TFIIS siRNA treated cells. The mRNA data might suggest that MCF10A did not respond as dramatically to TFIIS siRNA because it maintained a higher level of TFIIS mRNA. However, relative protein levels for TFIIS were lower in MCF10A than in MCF7.

**Figure 5:**
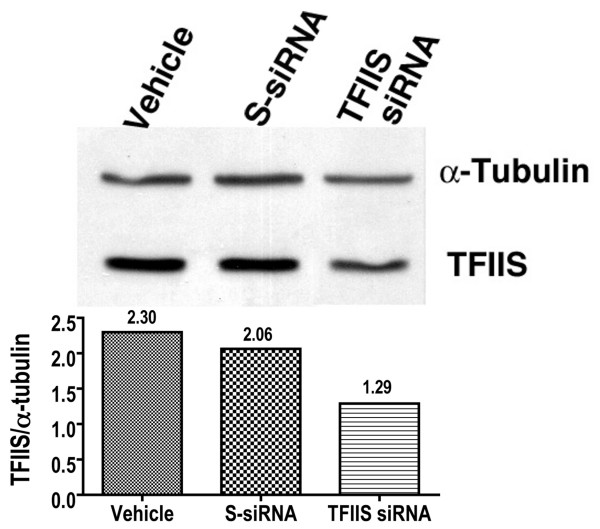
**Western analysis indicates reduced TFIIS protein after TFIIS siRNA treatment**. For the Western analysis, MCF7 cells were treated with vehicle, S-siRNA or TFIIS siRNA#2 as indicated. TFIIS and alpha-tubulin monoclonal antibodies were employed to detect their respective proteins. The ratio of TFIIS to alpha-tubulin as determined by imagequant (BioRad) is depicted by the bar-graph and listed as well above the columns. Cells were collected 48 hours after initial siRNA treatment.

Although the high degree of MCF7 cell death caused by TFIIS siRNA limited the amount of protein attainable, starting from large quantities of cells, we were able to perform a western analysis of TFIIS employing tubulin for normalization (Figure 5). The western indicated a 53% reduction in the amount TFIIS protein in TFIIS siRNA#2 treated cells. In S-siRNA-treated cells, TFIIS protein was 90% of control indicating that off-target effects of S-siRNA on TFIIS are minimal. The western data roughly conforms to the level of reduction of TFIIS in the C-ELISA assay.

**Figure 6:**
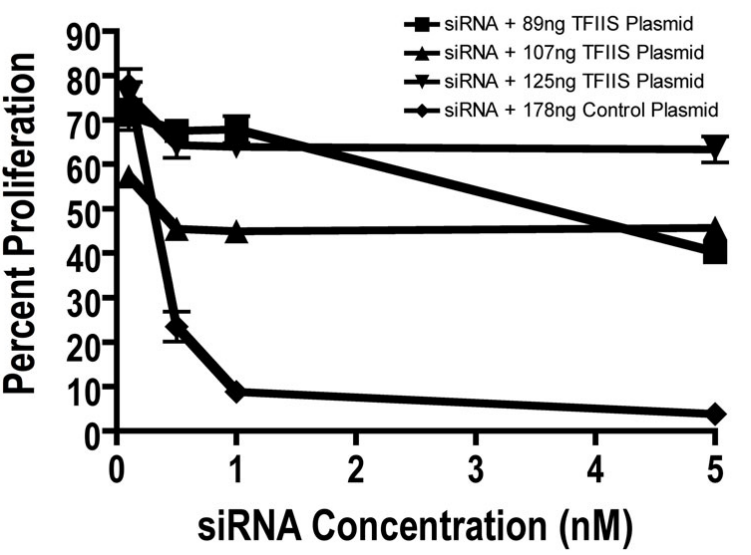
**TFIIS Recovery Assay**. The TFIIS mammalian expression vector pCMV6-XL5 was purchased from Origene. MCF7 cells were co-transfected with 89, 107 and 125 ng of the TFIIS:pCMV6-XL5 plasmid and 0.1, 0.5, 1.0, 5.0 nM TFIIS siRNA. In addition, 178 ng of a control pCMV6-XL5 plasmid with no TFIIS insert was transfected with 0.1, 0.5, 1.0 and 5.0 nM TFIIS siRNA. For control, 100% proliferation was set as proliferation of cells treated with vehicle + plasmid alone (without siRNA) at the relevant concentrations. Data points represent mean and standard error. Cell proliferation was determined 72 hours after initial transfection of siRNA and expression vector.

Results from a "recovery" assay indicate that the reduction of TFIIS mRNA was specifically responsible for MCF7 cell death (Figure 6). In the assay, a human TFIIS expression plasmid (TFIIS in pCMV-XL5; OriGene, Rockville, MD) was co-transfected with TFIIS siRNA to increase TFIIS mRNA. If inhibition of cell proliferation was a result of TFIIS mRNA knockdown, then additional TFIIS mRNA from the expression plasmid should recover cell proliferation. Indeed, the addition of the TFIIS expression plasmid resulted in a dramatic rescue of cell proliferation, indicating that inhibition of proliferation was a specific result of TFIIS knockdown.

### TFIIS knockdown activates P53 and c-myc in MCF7 cells

**Figure 7:**
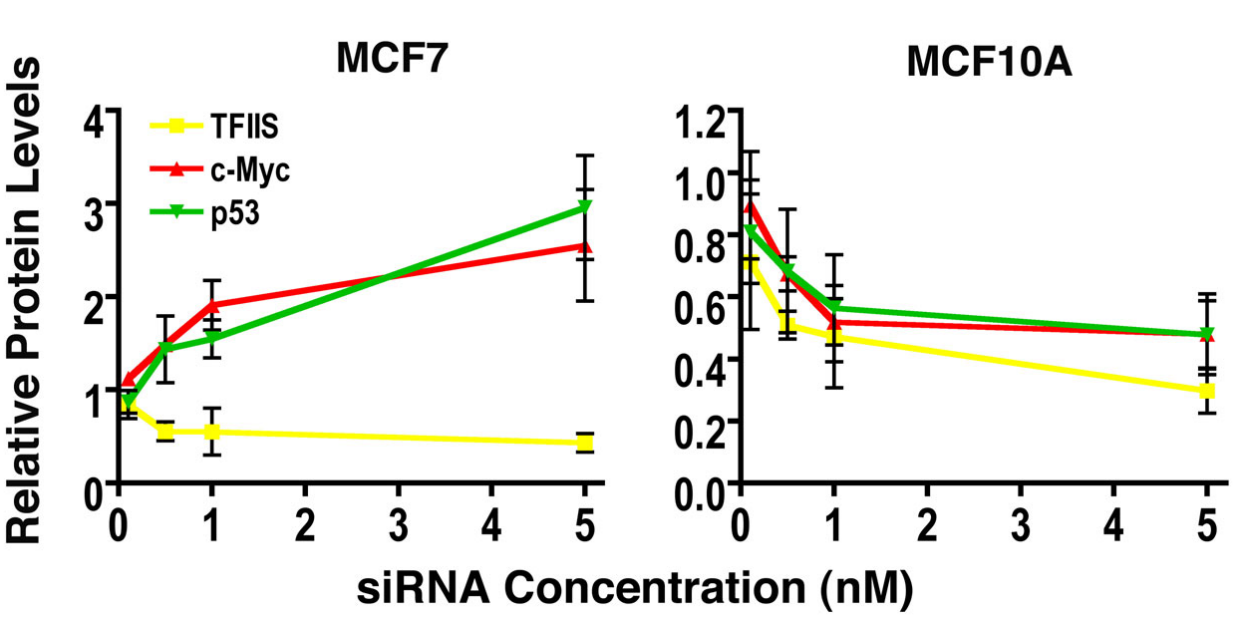
**Relative protein levels of TFIIS, c-myc and p53 were determined in MCF7 and MCF10A cells**. Protein values were determined for TFIIS siRNA #2 (Table 1) relative to GAPDH protein levels (TFIIS, c-myc or P53/GAPDH) and normalized with protein levels similarly determined from control S-siRNA treated cells. siRNA concentrations from 0.1 to 5.0 nM and standard error are indicated for each siRNA concentration. Data points represent mean and standard error and the assay performed 72 hours after initial siRNA transfection.

We employed a C-ELISA assay to assess relative amounts of TFIIS, c-myc and p53 in TFIIS siRNA treated MCF7 and MCF10A cells (Figure 7). GAPDH protein was employed as a baseline and relative levels of TFIIS, c-myc and p53 were determined. TFIIS siRNA reduced relative levels of TFIIS in both cancerous MCF7 and non-cancerous MCF10A cells in a dose dependent manner (Figure 7). However, MCF7 displayed a dose dependent increase of both c-myc and p53 of greater than 2.5-fold at 5 nM TFIIS siRNA. This was not the case for MCF10A cells, where both c-myc and p53 displayed a modest decrease in protein levels.

### Gene Array analysis of TFIIS siRNA in MCF7 and MCF10A

To gain further insight on effects of TFIIS siRNA on MCF7 and MCF10A cells, gene array studies were performed. The cDNA probes were from either 5 nM TFIIS siRNA treated MCF7 or MCF10A cells, and control cDNAs were from S-siRNA treated MCF7 or MCF10A cells. The experimental design allows for determining altered expression in MCF7 and MCF10A cells compared to expression in 5 nM S-siRNA treated cells (for experimental design, see table 2). Of the 16,659 70-mers, 58 genes (focus genes) displayed TFIIS knockdown specific expression changes unique to MCF10A and 49 unique focus genes were disclosed for MCF7, with one focus gene in common [see additional file 1].

**Figure 8:**
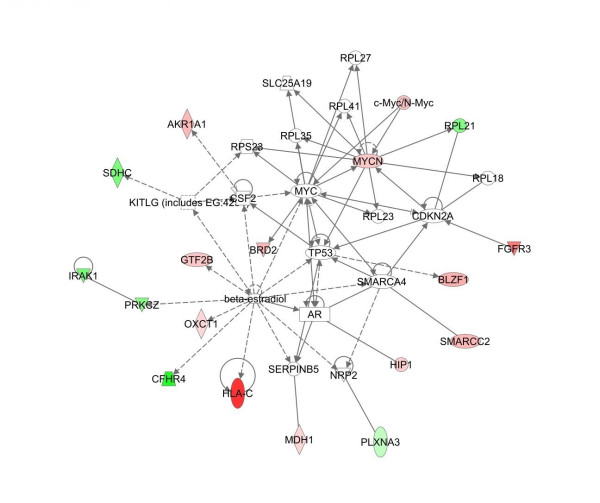
**Major pathways affected by TFIIS knockdown in MCF7**. Network analysis employed Ingenuity^® ^Systems software [31]. The software manufacturer maintains a copyright on the image networks. The Ingenuity networks display in color focus genes that are upregulated (green) and downregulated (red) by other proteins. The affect can be direct (solid lines) or indirect (broken lines). Triangle's represent kinases, diamonds enzymes, ovals Transcription regulators, rectangles G–Protein Coupled receptors and circles, other. The pathway represents MCF7 pathway 1. A list of focus genes for each pathway is provided in Additional file 1.

**Figure 9:**
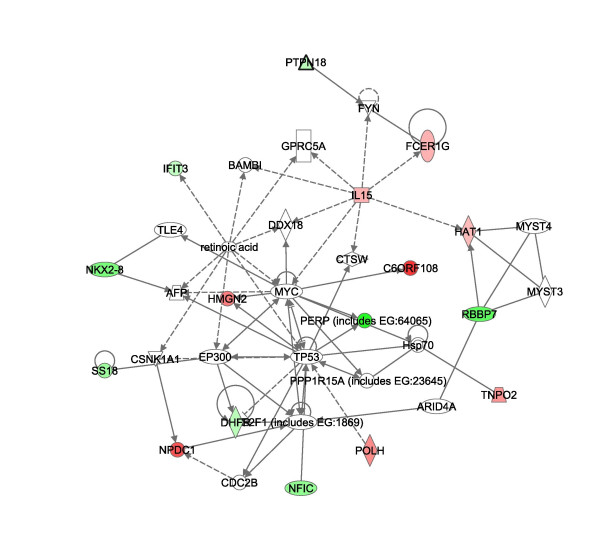
**MCF10A Pathway 1 affected by TFIIS knockdown**. The figure represents MCF10A pathways 1 derived from the Ingenuity^® ^Systems program, which maintains a copyright on the image networks. Labeling of the network is similar to that of figure 8.

**Figure 10:**
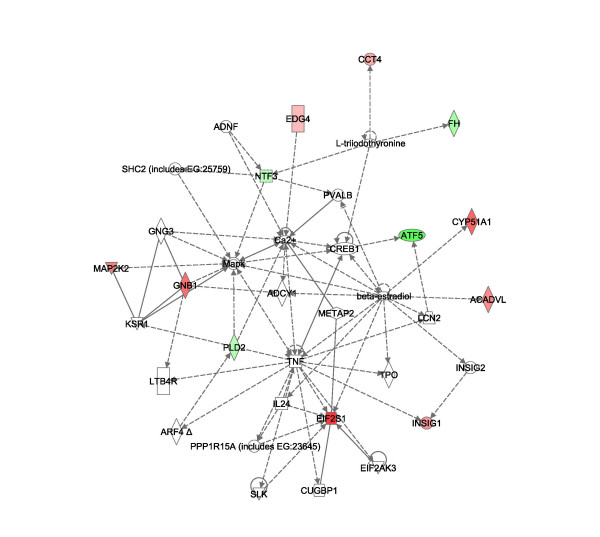
**MCF10A Pathway 2 affected by TFIIS knockdown**. The figure represents MCF10A pathways 2 derived from the Ingenuity^® ^Systems program, which maintains a copyright on the image networks. Labeling of the network is similar to that of figure 8.

Pathway analysis of the significant gene lists from above were imported into Ingenuity^® ^software (Figures 8, 9, 10, [31]). Pathways consist of a network of genes or proteins that directly or indirectly affect each other. Focus genes from the array were mapped to their corresponding gene in the Ingenuity Pathways. Ingenuity Pathway scores are derived from a p-value indicating the likelihood of the focus genes being found together due to random chance. A score of 2 indicates that there is a 1 in 100 chance that the focus genes are together in a network due to random chance. The top MCF7 pathways had scores of 40 and 15 and the top MCF10A pathways had scores of 37 and 25. The remaining pathways had scores of 2 and 3, and were not employed in our analysis to assure for highly significant data. For illustration purposes, pathways 1 of MCF7 (figure 8) and 1 and 2 of MCF10A are presented (Figures 9 and 10 respectively) with genes of the highly significant pathways listed in Additional file 1.

Some major "hubs" affected by TFIIS siRNA as disclosed in the pathway analysis include c-myc, p53, and estradiol for both MCF7 and MCF10A and the mitogen-activated protein kinase (MAPK) signaling pathway in MCF10A alone.

## Discussion

### Use of siRNA for studying effects of protein knockdown for cancer therapy

siRNA was employed to study the effects of TFIIS knockdown on cancer cells. Several lines of evidence indicate that siRNA methodologies employed for the analysis of TFIIS were valid. These include the effectiveness of the positive Eg5 control, the relatively limited effect of S-siRNA and the similar effects of three different TFIIS siRNAs directed at different regions of the TFIIS mRNA. Regarding Eg5, its inhibition of proliferation reached maximal effects at the low concentration of 0.5 nM siRNA in all cell lines (figures 1 and 3) so that transfection efficiencies in the different cell lines are comparable. TFIIS siRNA also induced a reduction in TFIIS mRNA and protein. Finally, recovery of proliferation reduced by TFIIS siRNA is attained by the addition of a TFIIS expression vector.

At the 5 nM TFIIS siRNA concentration, the reduction of mRNA and protein was not complete, nor was it expected to be. Even higher siRNA concentrations do not always allow for absolute protein or mRNA reduction. For example, siRNA reduction of Akt-1 was reported to be 51 and 73% effective for 20 and 80 nM siRNA, respectively [32]. In general, siRNA concentrations employed for knockdown by others are higher than those employed in the current study (20–100 nM) [32-34]. In our case, it was not necessary, nor possible to increase siRNA levels beyond 5 nM as we had nearly complete elimination of the MCF7 cells and significant reduction in proliferation of other cancer types. Furthermore, increasing siRNA levels is accompanied by increased nonspecific effects known as "off target effects" [32,35-37].

### Pathways affected by TFIIS knockdown

c-myc and p53 were major "hubs" in the TFIIS knockdown pathway for both MCF7 and MCF10A (pathway 1 of MCF7, figure 8 and pathway 1 of MCF10A, figure 9), in accord with the C-ELISA data of altered protein levels of p53 and c-myc. c-myc itself was not represented on the gene array and the p-value for p53 did not match our statistical criteria. However, increased c-myc expression in MCF7 and decreased c-myc expression in MCF10A fits well with the expression pattern of focus genes on the array. For example, in MCF7 arrays, downregulation of BRD2 and MYCN is in accord with c-myc repression of both genes [38,39]. Regarding MCF10A, the modest decrease in c-myc observed by the C-ELISA assay concurs with a decrease in expression of HGMN2 and C6ORF108 both of which are induced by c-myc [38,40]. Decreased c-myc in MCF10A also agrees with an increase in PERP as c-myc protein represses expression of PERP [41]. IL15 induces c-myc expression and the MCF10A array results indicate a decrease in IL15 expression in full agreement with decreased myc protein [42,43]. As such the gene array data correlates well with the C-ELISA data indicating increased c-myc in MCF7 and decreased levels in MCF10A.

Another hub of genes affected by TFIIS knockdown common to both MCF7 and MCF10A was beta-estradiol (Figures 7A and 7C). TFIIS knockdown appears to mimic the effects estrogen has on gene expression. More specifically, estradiol induces expression of PRKCZ and CFHR4 [44,45] and both increase on our MCF7 TFIIS knockdown array. Estradiol also induces expression of c-myc [46] and p53 [47] concurring with increased protein levels of both in MCF7 cells. Regarding MCF10A, a decrease in CYP51A1, EIF2S1 [48], and ACADVL [48] are all in accord with effects of estrogen on gene expression. However, estradiol also increases c-myc and p53 expression and in contrast MCF10A had moderately reduced levels of both proteins. In addition, within the estradiol expression pathways of MCF7 and MCF10A, different focus genes were affected [see Additional file 1]. It appears that TFIIS knockdown affects some similar pathways including c-myc/p53 and estradiol in both cell lines, though the effect on the particular expression of genes in the pathways differs and may contribute to the dissimilar consequences of TFIIS knockdown.

Another difference between MCF7 and MCF10A was the reduced expression of some components in the mitogen-activated protein kinase (MAPK) signaling pathway in MCF10A alone, as a result of TFIIS knockdown. For example, EDG4, a member of the G-coupled protein receptor family was downregulated. GNB1 a G-protein Ras signaling transducer and Map2K2 (MEK2), which phsophorylates MapK, displayed reduced-expression as well. PLD2 (phsopholipase D2) expression was increased in the TFIIS-knockdown MCF10A array. PLD2 is known to be involved in EGF receptor internalization and degradation [49]. As such it appears that TFIIS activates expression in a manner consistent with estradiol pathways in both MCF10A and MCF7 and reduces signal transduction pathways in MCF10A cells.

### TFIIS knockdown and the induction of cancer cell death

TFIIS knockdown is likely to set into effect a cascade, which modifies the level of expression of specific genes. Possible routes to invoke cancer cell death involve repression of oncogenes or activation of tumor suppressors. For example, it was previously shown that c-myc and c-fos oncogenes contain poly T stretches that pause or arrest transcription *in vitro *[14] and that those sites are TFIIS responsive [15,50-52]. As such we originally thought that TFIIS knockdown would decrease c-myc expression. Our observed increase in c-myc levels in MCF7 cells suggests that *in vitro *assays do not necessarily mirror *in vivo *effects. Other transcription elongation factors such as ELL, Elongin and TFIIF may suppress arresting at intrinsic sites *in vivo *[53] so that TFIIS may not necessarily play the key and prominent role of regulating c-myc elongation. Furthermore, although loss of c-myc activity is associated with tumor cell death [54], overexpression of c-myc can also be proapoptotic [55-58].

Another possibility is that TFIIS knockdown could alter transcription patterns and induce expression of a tumor suppressor gene. In this regard, inhibition of RNA Polymerase II has been implicated as resulting in activation of the p53 tumor suppressor [59,60], which is known to induce tumor regression [61]. Although TFIIS knockdown is not synonymous with RNAP inhibition, p53 could play a role in MCF7 cell death. Our data indicates that TFIIS knockdown does result in increased levels of p53 in MCF7 but not in MCF10A.

An additional pathway affected by TFIIS siRNA in both MCF7 and MCF10A was the β-estradiol hub, where TFIIS knockdown appears to mimic the activation of genes by estradiol. Although estrogen may be present in culture media, it was unlikely that the estrogenic pathway disclosed by the Ingenuity pathway software resulted from estrogen in the media as MCF10A does not express the estrogen receptor. In addition, identical conditions of cell growth (including any estrogen present in media) were employed for both control S-siRNA and TFIIS-siRNA treated cells. As such all pathways including the estrogenic pathway disclosed by the Ingenuity software can only be attributed to effects of TFIIS siRNA.

In addition to the increased p53 and c-myc in MCF7 cells, the induction of an estradiol "transcriptional overdose", may have ultimately contributed to MCF7 cell death. When breast cancer cells, including MCF7, are deprived of estrogen, they can develop estrogen hypersensitivity so that the addition of estrogen induces apoptosis [62,63]. Recent clinical considerations in treating estrogen refractory breast cancer includes use of estrogen on the estrogen hyper-sensitized cancer cells in post-menopausal women [62]. In MCF10A cells, the expression snapshot differs in some ways from MCF7. The activation of different focus genes in the estrogenic pathway, along with the moderate reduction of c-myc and p53, and reduced expression of components of the MapK signal transduction pathway are all likely to have been a determinant in its greater resistance to TFIIS siRNA.

TFIIS is primarily an elongation factor and TFIIS knockdown should primarily affect the efficiency of actively transcribed genes rather than activating new pathways. The β-estradiol hub is likely to be one of the active pathways in MCF7 and MCF10A cell lines of breast origin. This may in part explain why only one common focus gene was disclosed on the gene array. Only genes actively expressed in both MCF7 and MCF10A that contain TFIIS responsive arrest sites would be disclosed on the array. It is possible that there were other common focus genes in common between MCF7 and MCF10A that were masked by off target affects of the S-siRNA control. For example, siRNA itself causes some degree of Caspase 3/7 activation so that genes in the apoptotic pathway may not be observed.

### TFIIS as a possible target for therapeutics for breast and other Cancers

Aberrant gene expression is a hallmark of cancer cells. We propose that a further insult to the aberrant expression in cancer cells could cause cancer cell death with limited effects on normal cells, which maintain healthy transcription. The goal is to alter rather than inhibit transcription. Indeed, extended inhibition of RNAP is likely to be toxic as alpha-amanitin, an RNAP inhibitor from the mushroom genus Amanita, is fatal if ingested [64].

For a cancer target to be justified, its inhibition must not only eradicate cancer cells, but should also be tolerated on the cellular and physiological levels. Data from clinical trials of HDAC inhibitors and Flavopiridol, both of which effect transcription mechanisms, indicates a degree of physiological tolerance to transcription-modifying agents [13,65]. *In vivo *knockout experiments also confirm that TFIIS depletion may be tolerated on the cellular and physiological levels. In yeast TFIIS knockout strains are viable [66,67]. In TFIIS knockout mice, TFIIS had a critical role in definitive haematopoiesis and embryos did not come to term because they were anemic [68]. However, TFIIS knockout did not prevent cell growth, differentiation or development and embryos showed similar overall organ development until E13.5 when the lack of terminal differentiation and red ghosts took its toll [68].

The inhibition of haematopoiesis in mice by TFIIS knockout should not deter further study of TFIIS as a cancer target. First, even if TFIIS knockout inhibits haematopoiesis, red blood ghosts already exist in an adult. In addition, current chemotherapy protocols can employ agents that damage haematopoeisis (along with other cells that rapidly divide), and can be treated with transfusions in severe cases. Second, in knockout mice, the lack of TFIIS is complete and exists since inception. In our study, the reduction of TFIIS was not complete, yet was sufficient to induce dramatic cell death in the MCF7 cell line. Finally, RNAP arrests at specific DNA sequences whereupon TFIIS exerts its role in reinitiating transcription. There exists some degree of sequence difference between mouse and human DNA so that a similar inhibition of haematopoiesis in humans needs to be confirmed.

In the case of TFIIS, the non-cancerous MCF10A cell line was dramatically less affected than MCF7 cells. MCF10A cells were employed as a control for cancer cells because they are not tumorigenic and have been used as a "normal" control by others [1]. However, it is unlikely that MCF10A truly represents a "normal" cell. MCF10A does display genomic alterations, modified expression of some genes such as ERK1, and does grow in 3-D semi-solid medium [69,70]. This may explain why there is no difference in proliferation between the PL45 cells and the MCF10A cells and minimal (only at low siRNA concentrations) differences between A549 and MCF10A. It is likely that transcription in most immortalized cells is aberrant so that targeting the transcription apparatus in immortalized cells may also affect cell viability.

## Conclusion

Our data suggests that further studies assessing transcription factors and specifically TFIIS for cancer therapy are called for. At the core of our reasoning are the minimal effects of S-siRNA on cancer cell proliferation and the effective inhibition of proliferation by siRNA directed at TFIIS. This is in full accord with effects of the well-studied anticancer agent flavopiridol, which induces apoptosis and has recently been shown to act by inhibition of transcription elongation [71,72]. However, we also draw attention to an inherent challenge in cell culture analysis of transcription factor knockdown. Cell lines are unlikely to present "normal" transcription so that targeting transcription in culture may not allow for a complete picture of the effectiveness of targeting the transcription machinery. However, siRNA and cell culture may provide for an initial indication of transcription factors such as TFIIS that should be targeted for further investigation.

## Competing interests

The authors declare that they have no competing interests.

## Authors' contributions

KH carried out all the experiments described alone, except for the gene array. JC performed the gene array experiments and analysis together with KH and under the direct supervision of RKP. AG was responsible for the overall project design and implementation. The manuscript was drafted by all parties, reviewed and revised by both FDA and University of Maryland research teams.

## Pre-publication history

The pre-publication history for this paper can be accessed here:



## Supplementary Material

Additional file 1 A list of focus genes from the gene array. Click here for file
