# Supplementary material for: Knockdown of TFIIS by RNA silencing inhibits cancer cell proliferation and induces apoptosis
Source: BMC Cancer. 2008 May 12;8:133. doi: 10.1186/1471-2407-8-133 (PMC2390572; doi:10.1186/1471-2407-8-133)
Supplement: Additional file 1 — A list of focus genes from the gene array. [file 1471-2407-8-133-S1.doc]

**Determination of GAPDH as a control for C-ELISA Experiments.** *MTS assay results, which correlate directly with cell number, plotted against the Relative Light units (RLU) from the GAPDH expression after a C-ELISA experiment.*

Appendix 1: MCF10A

| GENEBANKACC | GENENAME | LOCUSID | FOLD | PVALUE | RANKING_ORDER |
| --- | --- | --- | --- | --- | --- |
| L46590 | ACADVL--acyl-Coenzyme A dehydrogenase, very long chain (ACADVL), nuclear gene encoding mitochondri | 37 | 1.566 | 0.0422 | 1 |
| U23942 | CYP51A1--cytochrome P450, family 51, subfamily A, polypeptide 1 (CYP51A1), mRNA. | 1595 | 1.8053 | 0.0312 | 2 |
| NM_000791 | DHFR--dihydrofolate reductase (DHFR), mRNA. | 1719 | 0.7108 | 0.0018 | 3 |
| Y15718 | DTNB--Dystrobrevin, beta | 1838 | 0.748 | 0.0497 | 4 |
| J02645 | EIF2S1--eukaryotic translation initiation factor 2, subunit 1 alpha, 35kDa (EIF2S1), mRNA. | 1965 | 2.1485 | 0.0416 | 5 |
| M33195 | FCER1G--Fc fragment of IgE, high affinity I, receptor for; gamma polypeptide (FCER1G), mRNA. | 2207 | 1.3454 | 0.0211 | 6 |
| U59309 | FH--fumarate hydratase (FH), nuclear gene encoding mitochondrial protein, mRNA. | 2271 | 0.64 | 0.0099 | 7 |
| X04526 | GNB1--guanine nucleotide binding protein (G protein), beta polypeptide 1 (GNB1), mRNA. | 2782 | 1.7475 | 0.0415 | 8 |
| X13546 | HMGN2--high-mobility group nucleosomal binding domain 2 (HMGN2), mRNA. | 3151 | 1.6602 | 0.0241 | 9 |
| AF026939 | IFIT3--interferon-induced protein with tetratricopeptide repeats 3 (IFIT3), mRNA. | 3437 | 0.7425 | 0.0406 | 10 |
| AF031167 | IL15--interleukin 15 (IL15), transcript variant 3, mRNA. | 3600 | 1.3552 | 0.0168 | 11 |
| U96876 | INSIG1--insulin induced gene 1 | 3638 | 1.4891 | 0.0334 | 12 |
| AJ224741 | MATN3--matrilin 3 (MATN3), mRNA. | 4148 | 0.6045 | 0.026 | 13 |
| U37707 | MPP3--membrane protein, palmitoylated 3 (MAGUK p55 subfamily member 3) (MPP3), mRNA. | 4356 | 0.7429 | 0.0469 | 14 |
| D32002 | NCBP1--nuclear cap binding protein subunit 1, 80kDa (NCBP1), mRNA. | 4686 | 1.4207 | 0.0353 | 15 |
| X12492 | NFIC--nuclear factor I/C (CCAAT-binding transcription factor) (NFIC), transcript variant 1, mRNA. | 4782 | 0.5787 | 0.0325 | 16 |
| X53655 | NTF3--neurotrophin 3 (NTF3), mRNA. | 4908 | 0.7229 | 0.0374 | 17 |
| AF038440 | PLD2--phospholipase D2 (PLD2), mRNA. | 5338 | 0.6951 | 0.0182 | 18 |
| AB024313 | POLH--polymerase (DNA directed), eta (POLH), mRNA. | 5429 | 1.5674 | 0.0347 | 19 |
| L11285 | MAP2K2--mitogen-activated protein kinase kinase 2 (MAP2K2), mRNA. | 5605 | 1.6386 | 0.039 | 20 |
| X72841 | RBBP7--retinoblastoma binding protein 7 (RBBP7), mRNA. | 5931 | 0.4489 | 0.0481 | 21 |
| M95549 | SLC5A2--solute carrier family 5 (sodium/glucose cotransporter), member 2 (SLC5A2), mRNA. | 6524 | 0.5758 | 0.0024 | 22 |
| X79201 | SS18--synovial sarcoma translocation, chromosome 18 (SS18), transcript variant 2, mRNA. | 6760 | 0.6152 | 0.049 | 23 |
| AF030424 | HAT1--histone acetyltransferase 1 (HAT1), mRNA. | 8520 | 1.3065 | 0.0264 | 24 |
| AF011466 | EDG4--endothelial differentiation, lysophosphatidic acid G-protein-coupled receptor, 4 (EDG4), mRNA. | 9170 | 1.3014 | 0.0449 | 25 |
| AF026291 | CCT4--chaperonin containing TCP1, subunit 4 (delta) (CCT4), mRNA. | 10575 | 1.3713 | 0.0475 | 26 |
| AF040105 | C6orf108--chromosome 6 open reading frame 108 (C6orf108), transcript variant 1, mRNA. | 10591 | 2.2072 | 0.0336 | 27 |
| U92642 | GPR45--G protein-coupled receptor 45 | 11250 | 0.752 | 0.0411 | 28 |
| AL136773 | PTK9L--PTK9L protein tyrosine kinase 9-like (A6-related protein) (PTK9L), mRNA. | 11344 | 1.5391 | 0.0213 | 29 |
| AB021663 | ATF5--activating transcription factor 5 (ATF5), mRNA. | 22809 | 0.4392 | 0.0289 | 30 |
| AF000297 | NKX2-8--NK2 transcription factor related, locus 8 (Drosophila) (NKX2-8), mRNA. | 26257 | 0.5228 | 0.0122 | 32 |
| X79568 | PTPN18--protein tyrosine phosphatase, non-receptor type 18 (brain-derived) (PTPN18), mRNA. | 26469 | 0.7099 | 0.035 | 33 |
| AK022217 | Homo sapiens cDNA FLJ12155 fis, clone MAMMA1000472 | 30000 | 1.5035 | 0.0358 | 34 |
| NM_015971 | MRPS7--mitochondrial ribosomal protein S7 (MRPS7), nuclear gene encoding mitochondrial protein, mR | 51081 | 1.3204 | 0.0212 | 35 |
| AK000403 | CKLFSF6--chemokine-like factor super family 6 (CKLFSF6), mRNA. | 54918 | 0.5753 | 0.0157 | 36 |
| AL050221 | NPDC1--neural proliferation, differentiation and control, 1 (NPDC1), mRNA. | 56654 | 1.9644 | 0.0311 | 37 |
| NM_020156 | C1GALT1--core 1 UDP-galactose:N-acetylgalactosamine-alpha-R beta 1,3-galactosyltransferase (C1GALT | 56913 | 0.4947 | 0.0271 | 38 |
| AB007961 | KIAA0492--KIAA0492 protein | 57238 | 1.5644 | 0.0396 | 39 |
| AB037814 | KIAA1393--KIAA1393 | 57570 | 2.0088 | 0.0289 | 40 |
| NM_022121 | PERP--PERP, TP53 apoptosis effector (PERP), mRNA. | 64065 | 0.3344 | 0.006 | 41 |
| NM_024688 | C10orf68--chromosome 10 open reading frame 68 (C10orf68), mRNA. | 79741 | 1.3757 | 0.0185 | 42 |
| NM_024943 | FLJ23235--hypothetical protein FLJ23235 (FLJ23235), mRNA. | 80008 | 0.7656 | 0.0363 | 43 |
| AL136566 | C9orf58--chromosome 9 open reading frame 58 (C9orf58), transcript variant 1, mRNA. | 83543 | 0.6043 | 0.0413 | 44 |
| AL137544 | C6orf157--Chromosome 6 open reading frame 157 | 90025 | 0.5489 | 0.0242 | 45 |
| AF161451 | NSE1--non-SMC (structural maintenance of chromosomes) element 1 protein (NSE1), mRNA. | 197370 | 1.3343 | 0.0359 | 46 |
| AY014272 | Similar to FKSG30 | 440915 | 1.7689 | 0.0417 | 47 |
| U93305 | SYP--synaptophysin (SYP), mRNA. | | 0.7477 | 0.0468 | 48 |
| L32140 | AFM--afamin (AFM), mRNA. |  | 1.6878 | 0.0001 | 49 |
| Positive control | HK--Beta-actin |  | 1.3569 | 0.0404 | 50 |
| AL136226 | Human DNA sequence from clone RP3-486D24 on chromosome 6 Contains a ribosomal protein L7A (RPL7A) ps | | 2.727 | 0.0436 | 51 |
| AC002400 | NDUFAB1--NADH dehydrogenase (ubiquinone) 1, alpha/beta subcomplex, 1, 8kDa (NDUFAB1), mRNA. | | 2.0065 | 0.0109 | 52 |
| AL022721 | RPL10A--ribosomal protein L10a (RPL10A), mRNA. | | 2.534 | 0.0115 | 53 |
| XM_013105 | Homo sapiens similar to laminin receptor 1 (67kD, ribosomal protein SA) (H. sapiens) (LOC65910), mRN | | 2.3607 | 0.0185 | 54 |
| AL117577 | MRNA; cDNA DKFZp434C108 (from clone DKFZp434C108) | | 0.4171 | 0.0492 | 55 |
| XM_011219 | Homo sapiens similar to ribosomal protein S8 (H. sapiens) (LOC65447), mRNA | | 1.8302 | 0.038 | 56 |
| AC005390 | Homo sapiens chromosome 19, cosmid R31180 | | 0.5085 | 0.0443 | 57 |
| NM_005579 | ZXDB--zinc finger, X-linked, duplicated B (ZXDB), mRNA. | | 0.6353 | 0.0395 | 58 |
| AL121845 | ZGPAT--zinc finger, CCCH-type with G patch domain (ZGPAT), transcript variant 3, mRNA. | | 0.4878 | 0.0139 | 59 |

Appendix 2. MCF7

| GENEBANKACC | GENENAME | LOCUSID | FOLD | PVALUE | RANKING_ORDER |
| --- | --- | --- | --- | --- | --- |
| M62762 | ATP6C--ATPase, H+ transporting, lysosomal (vacuolar proton pump) 16kD | 527 | 0.6741 | 0.0397 | 1 |
| M64347 | FGFR3--fibroblast growth factor receptor 3 (achondroplasia, thanatophoric dwarfism) (FGFR3), trans | 2261 | 3.1667 | 0.0241 | 2 |
| NM_001514 | GTF2B--general transcription factor IIB (GTF2B), mRNA. | 2959 | 1.5955 | 0.0021 | 3 |
| NM_001515 | GTF2H2--general transcription factor IIH, polypeptide 2, 44kDa (GTF2H2), mRNA. | 2966 | 2.0101 | 0.0385 | 4 |
| AF052288 | HIP1--huntingtin interacting protein 1 (HIP1), mRNA. | 3092 | 1.4912 | 0.0196 | 5 |
| X58536 | HLA-C--major histocompatibility complex, class I, C (HLA-C), mRNA. | 3107 | 5.7696 | 0.0485 | 6 |
| L76191 | IRAK1--interleukin-1 receptor-associated kinase 1 (IRAK1), mRNA. | 3654 | 0.5425 | 0.0158 | 7 |
| D55654 | MDH1--malate dehydrogenase 1, NAD (soluble) (MDH1), mRNA. | 4190 | 1.4098 | 0.0148 | 8 |
| M55330 | MYCNOS--V-myc myelocytomatosis viral related oncogene, neuroblastoma derived (avian) opposite strand | 4613 | 1.6013 | 0.0128 | 9 |
| U62961 | OXCT1--3-oxoacid CoA transferase 1 (OXCT1), nuclear gene encoding mitochondrial protein, mRNA. | 5019 | 1.3815 | 0.0135 | 10 |
| Z15108 | PRKCZ--protein kinase C, zeta (PRKCZ), mRNA. | 5590 | 0.5941 | 0.007 | 11 |
| AL050268 | RAB1A--RAB1A, member RAS oncogene family (RAB1A), mRNA. | 5861 | 0.5055 | 0.042 | 12 |
| D42040 | BRD2--bromodomain containing 2 (BRD2), mRNA. | 6046 | 1.8478 | 0.0441 | 13 |
| U25789 | RPL21--ribosomal protein L21 (RPL21), mRNA. | 6144 | 0.5787 | 0.0137 | 14 |
| U57877 | SDHC--succinate dehydrogenase complex, subunit C, integral membrane protein, 15kDa (SDHC), nuclear | 6391 | 0.56 | 0.0233 | 15 |
| U66616 | SMARCC2--SWI/SNF related, matrix associated, actin dependent regulator of chromatin, subfamily c, | 6601 | 1.8242 | 0.0372 | 16 |
| D28588 | SP2--Sp2 transcription factor (SP2), mRNA. | 6668 | 1.3695 | 0.0155 | 17 |
| X05615 | TG--thyroglobulin (TG), mRNA. | 7038 | 1.9047 | 0.0343 | 18 |
| X06956 | TUBA1--tubulin, alpha 1 (testis specific) (TUBA1), mRNA. | 7277 | 1.4804 | 0.035 | 19 |
| Y08614 | XPO1--exportin 1 (CRM1 homolog, yeast) (XPO1), mRNA. | 7514 | 0.6233 | 0.0361 | 20 |
| U09368 | ZNF140--zinc finger protein 140 (clone pHZ-39) (ZNF140), mRNA. | 7699 | 1.8488 | 0.0056 | 21 |
| AB012911 | FZD6--frizzled homolog 6 (Drosophila) (FZD6), mRNA. | 8323 | 0.5644 | 0.0486 | 22 |
| U79751 | BLZF1--Basic leucine zipper nuclear factor 1 (JEM-1) | 8548 | 1.956 | 0.0194 | 23 |
| AF102166 | CLIC3--chloride intracellular channel 3 (CLIC3), mRNA. | 9022 | 0.4661 | 0.0482 | 24 |
| AJ132583 | NPEPPS--aminopeptidase puromycin sensitive (NPEPPS), mRNA. | 9520 | 0.4699 | 0.002 | 25 |
| AB007896 | KIAA0436--Putative prolyl oligopeptidase | 9581 | 0.5625 | 0.0349 | 26 |
| AK000709 | Homo sapiens cDNA FLJ20702 fis, clone KAIA2174 | 9871 | 2.7215 | 0.0087 | 27 |
| AK001703 | AMMECR1--Hypothetical protein LOC286505 | 9949 | 0.7313 | 0.0096 | 28 |
| J04794 | AKR1A1--aldo-keto reductase family 1, member A1 (aldehyde reductase) (AKR1A1), transcript variant | 10327 | 1.8216 | 0.004 | 29 |
| X98337 | CFHL4--complement factor H-related 4 (CFHL4), mRNA. | 10877 | 0.374 | 0.0319 | 30 |
| NM_007071 | HHLA3--HERV-H LTR-associating 3 (HHLA3), mRNA. | 11147 | 0.7358 | 0.0184 | 31 |
| M81637 | GCA--grancalcin, EF-hand calcium binding protein (GCA), mRNA. | 25801 | 2.0324 | 0.0026 | 33 |
| AL122063 | GLTSCR2--Glioma tumor suppressor candidate region gene 2 | 29997 | 2.2913 | 0.0321 | 34 |
| NM_015933 | HSPC016--hypothetical protein HSPC016 (HSPC016), mRNA. | 51372 | 0.6662 | 0.0324 | 35 |
| X87852 | PLXNA3--plexin A3 (PLXNA3), mRNA. | 55558 | 0.7644 | 0.0282 | 36 |
| AK000693 | C9orf55--chromosome 9 open reading frame 55 (C9orf55), mRNA. | 55667 | 1.7595 | 0.0285 | 37 |
| NM_020188 | DC13--DC13 protein (DC13), mRNA. | 56942 | 0.5957 | 0.0355 | 38 |
| NM_020226 | PRDM8--PR domain containing 8 | 56978 | 1.6139 | 0.0279 | 39 |
| AB033107 | ZNF608--Zinc finger protein 608 | 57507 | 1.7792 | 0.0153 | 40 |
| AB033079 | TDE2--tumor differentially expressed 2 (TDE2), mRNA. | 57515 | 0.6003 | 0.0032 | 41 |
| NM_022489 | C14orf173--chromosome 14 open reading frame 173 (C14orf173), mRNA. | 64423 | 0.7056 | 0.0362 | 42 |
| NM_024719 | GRTP1--growth hormone regulated TBC protein 1 (GRTP1), mRNA. | 79774 | 0.5449 | 0.0184 | 43 |
| NM_024839 | RPP21--ribonuclease P 21kDa subunit (RPP21), mRNA. | 79897 | 0.5917 | 0.0318 | 44 |
| AF081192 | H2AFV--H2A histone family, member V (H2AFV), transcript variant 4, mRNA. | 94239 | 0.5319 | 0.0093 | 45 |
| NM_016415 | LOC51216--Clone FLB3816 | | 0.5693 | 0.033 | 46 |
| AL122072 | FAM36A--family with sequence similarity 36, member A (FAM36A), mRNA. | | 0.7282 | 0.039 | 47 |
| AK000309 | FLJ20302--hypothetical protein FLJ20302 | | 0.3634 | 0.0369 | 48 |
| AB016247 | SC5DL--sterol-C5-desaturase (ERG3 delta-5-desaturase homolog, fungal)-like (SC5DL), mRNA. | | 2.3238 | 0.0438 | 49 |
| AL137307 | PDZRN3--PDZ domain containing RING finger 3 | | 0.5314 | 0.0183 | 50 |

Appendix 3. Shared Genes

| GENEBANKACC | GENENAME | LOCUSID | FOLD | PVALUE |
| --- | --- | --- | --- | --- |
| NM_014341 | MTCH1--mitochondrial carrier homolog 1 (C. elegans) (MTCH1), nuclear gene encoding mitochondrial p | 23787 | 0.4652 | 0.028 |

**Appendix III.**

Ingenuity Networks and Associated Focus Genes

| MCF7 | molecules in network | score | focus genes | top functions |  |  |  |  | |  |
| --- | --- | --- | --- | --- | --- | --- | --- | --- | --- | --- |
| 1 | AKR1A1, AR, beta-estradiol, BLZF1, BRD2, c-Myc/N-Myc, CDKN2A, CFHR4, CSF2, FGFR3, GTF2B, HIP1, HLA-C, IRAK1, KITLG (includes EG:4254), MDH1, MYC, MYCN, NRP2, OXCT1, PLXNA3, PRKCZ, RPL18, RPL21, RPL23, RPL27, RPL35, RPL41, RPS23, SDHC, SERPINB5, SLC25A19, SMARCA4, SMARCC2, TP53 | 40 | 17 | Cell Death, Cancer, Cell Cycle | |  |  |  | |  |
| 2 | AGTR1B, ANP32A, AREG, ATP6V0C, CDH11, CDKN1C, CLU, CTNNB1, EGFR, FBN1, FOS, Frizzled, FZD6, GJA1, GTF2H2, HGF, IL1R1, MAP4, MAT2A, PAX8, PHB, PLK1, PTPRJ, RAB1A, RPLP2, RTK, SERPINH1, SFRP1, SP2, TCF7L2 (includes EG:6934), TG, TGFB1, TUBA1, TUBB, XPO1 | 15 | 8 | Cellular Development, Cellular Growth and Proliferation, Cancer | | | |  | |  |
| MCF10A | molecules in network | score | focus genes | top functions |  |  |  |  | |  |
| 1 | AFP, ARID4A, BAMBI, C6ORF108, CDC2B, CSNK1A1, CTSW, DDX18, DHFR, E2F1 (includes EG:1869), EP300, FCER1G, FYN, GPRC5A, HAT1, HMGN2, Hsp70, IFIT3, IL15, MYC, MYST3, MYST4, NFIC, NKX2-8, NPDC1, PERP (includes EG:64065), POLH, PPP1R15A (includes EG:23645), PTPN18, RBBP7, retinoic acid, SS18, TLE4, TNPO2, TP53 | 37 | 16 | Cellular Development, Immune and Lymphatic System Development and Function, Cancer | | | | | | |
| 2 | ACADVL, ADCY1, ADNF, ARF4, ATF5, beta-estradiol, Ca2+, CCT4, CREB1, CUGBP1, CYP51A1, EDG4, EIF2AK3, EIF2S1, FH, GNB1, GNG3, IL24, INSIG1, INSIG2, KSR1, L-triiodothyronine, LCN2, LTB4R, MAP2K2, Mapk, METAP2, NTF3, PLD2, PPP1R15A (includes EG:23645), PVALB, SHC2 (includes EG:25759), SLK, TNF, TPO | 25 | 12 | Cellular Growth and Proliferation, Cardiovascular Disease, Behavior | | | | |  | |
